# Ertapenem versus piperacillin/tazobactam for the treatment of complicated infections: a meta-analysis of randomized controlled trials

**DOI:** 10.1186/1471-2334-9-193

**Published:** 2009-12-02

**Authors:** Mao Mao An, Zui Zou, Hui Shen, Jun Dong Zhang, Meng Li Chen, Ping Liu, Rui Wang, Yuan Ying Jiang

**Affiliations:** 1 Department of Clinical Pharmacology, Chinese People's Liberation Army General Hospital, 28 Fuxing Road, Beijing 100853, PR China; 2 Department of Anesthesiology, Changzheng Hospital, Second Military Medical University, Shanghai, 200433, PR China; 3 Chinese Acad Med Sci, Peking Union Med Coll, Fuwai Hosp & Cardiovasc Inst, 167 Beilishi Road, Beijing 100037, PR China; 4 Department of Pharmacology, School of Pharmacy, Second Military Medical University, 325 Guo He Road, Shanghai 200433, PR China

## Abstract

**Background:**

Ertapenem, a new carbapenem with a favorable pharmacokinetic profile, has been approved for the treatment of complicated intra-abdominal Infections (cIAIs), acute pelvic infections (APIs) and complicated skin and skin-structure infections (cSSSIs). The aim of this study is to compare the efficacy and safety of ertapenem with piperacillin/tazobactam, which has been reported to possess good efficacy for the treatment of these complicated infections.

**Methods:**

We performed a meta-analysis of randomized controlled trials identified in PubMed, Cochrane library and Embase that compared the efficacy and safety of ertapenem with piperacillin/tazobactam for the treatment of complicated infections including cIAIs, APIs, cSSSIs. The primary efficacy outcome was clinical treatment success assessed at the test-of-cure visit. The primary safety outcome was drug related clinical and laboratory adverse events occurred during the treatment and the post-treatment period.

**Result:**

Six RCTs, involving 3161 patients, were included in our meta-analysis. Ertapenem was associated similar clinical treatment success with piperacillin/tazobactam for complicated infections treatment (clinically evaluable population, 1937 patients, odds ratios: 1.15, 95% confidence intervals: 0.89-1.49; modified intention to treat population, 2855 patients, odds ratios: 1.03, 95% confidence intervals: 0.87-1.22). All of secondary efficacy outcomes analysis obtained similar findings with clinical treatment success. No difference was found about the incidence of drug related adverse events between ertapenem and piperacillin/tazobactam groups.

**Conclusion:**

This meta-analysis provides evidence that ertapenem 1 g once a day can be used as effectively and safely as recommended dose of piperacillin/tazobactam, for the treatment of complicated infections, particularly of mild to moderate severity. It is an appealing option for the treatment of these complicated infections.

## Background

The treatment of complicated infections such as complicated intra-abdominal infections (cIAIs), acute pelvic infections (APIs) and complicated skin and skin-structure infections (cSSSIs) is a challenge for clinicians. They are most often polymicrobial or mixed infections which caused by pathogens involving a mixture of gram-positive and gram-negative aerobic and anaerobic organisms including staphylococcus, streptococci, enterococci, Enterobacteriaceae, anaerobic coccobacilli, anaerobic bacilli [1-4].

The outcome of complicated infections depends on the timely diagnosis and treatment which involves appropriate antimicrobial therapy directing to the residual infecting microorganisms. Absent or inadequate antibiotic therapy results in both increased failure rates and mortality. Empiric antimicrobial treatment of the complicated infection requires parenteral coverage of a broad spectrum of potential pathogens, often including β-lactam/β-lactamase inhibitor combination, carbapenems, cephalosporins with anaerobic coverage, or combination therapy consisting of a cephalosporin, fluoroquinolone, or aminoglycoside plus an anti-anaerobic agent [5-7].

Ertapenem (Merck & Co., Inc., USA), a long-acting and once-daily parenteral carbapenem, was approved for the treatment of complicated infections such as cIAIs, APIs, cSSSIs. It is rapidly bactericidal against most of the predominant intra-abdominal, skin/skin-structure and pelvic pathogens, including many that produce extended-spectrum or AmpC β-lactamase producing Enterobacteriaceae which are resistant to cephalosporins and β-lactam/β-lactamase inhibitor combination [8-10]. It is an appealing option for the treatment of these complicated infections not only because of its spectrum of antimicrobial activity, but also its convenient dosing schedule, and sounds a useful alternative for combination and/or multidosed antibiotic regimens for the empiric treatment.

Although ertapenem has limited activity *in vitro *against enterococci and *Pseudomonas aeruginosa *which may be encountered in cIAIs, APIs and cSSSIs, several randomized controlled trials (RCTs) suggested that ertapenem is at least as effective as piperacillin/tazobactam, a β-lactam/β-lactamase inhibitor combination agent that is routinely used in the treatment of these complicated infections [11-16]. Aiming to compare more conclusively the efficacy and safety of ertapenem with piperacillin/tazobactam in these complicated infections, we undertook a system review with meta-analysis of relevant RCTs.

## Methods

### Data sources

The study was done with a prespecified search strategy and study eligibility criteria. We did an extensive electronic search of PubMed (up to March 2009), the Cochrane Central Register of Controlled Trials (Cochrane Library Issue 1, 2009), and Embase (1980 to March 2009) to identify relevant RCTs for our meta-analysis, without language restrictions. We restricted the search to randomized controlled trials. Search term combinations were "ertapenem" "piperacillin/tazobactam" "polymicrobial infections" and similar, "mixed infections" and similar, "complicated intra-abdominal infections", "complicated skin and skin-structure infections", "acute pelvic infections". All reference lists from the relevant articles and reviews were hand searched for additional eligible studies. Experts in the field were also consulted. The articles that were not available to us were requested from the authors.

### Study selection

Two reviewers (MMA and ZZ) independently searched the literature and examined relevant RCTs for further assessment. Any study was included in our meta-analysis if it was a RCT; if it involved patients of all ages with cIAIs, APIs, cSSSIs or other complicated infections; if it compared the efficacy and safety of ertapenem with piperacillin/tazobactam; if it reported specific data regarding clinical treatment success, microbiological treatment success, mortality, adverse events. Trials with blinded and unblinded design were both included. Abstracts in scientific conferences were not included in the meta-analysis. Experimental trials and trials focusing on pharmacokinetic or pharmacodynamic variables were excluded.

### Qualitative assessment

Evaluation of the methodological quality of the RCTs included in the meta-analysis was performed independently by the two reviewers (MMA and ZZ) using the Jadad scoring system as follows: One point is awarded for the presence of randomization, blinding and data about study withdrawals respectively. Also, if the randomization or blinding procedures are appropriate, one point is awarded for each procedure; no points are awarded if no data are provided regarding the methodology of the above-mentioned procedures; finally, if any of these procedures is not deemed appropriate one point is deducted for each one [17]. The maximum score that can be attributed to an RCT is 5. An RCT with a score higher than 2 is considered as an RCT of adequately good quality [18,19].

### Data extraction

Two reviewers (MMA and ZZ) independently extracted data from included trials. Data were extracted from each study with a predesigned review form. In case of any disagreement between the two reviewers, a third reviewer extracted the data and results were attained by consensus. We contacted the authors of trials for missing data when necessary.

The following data were extracted from each study: year of publication, clinical setting, patient population, and number of patients (intention to treat [ITT], modified intention to treat [MITT], clinically and microbiologically evaluable populations), antimicrobial agents and doses used, concomitant antibacterial agents, clinical and microbiological outcomes, mortality and drug related adverse events. The MITT population was composed of randomized patients who received at least one dose of study drug and met the minimal disease definition. The clinically evaluable population comprised patients that fulfilled all inclusion and exclusion criteria in the individual RCTs, had complete follow-up, and for whom data on treatment outcomes were available but not indeterminate. The microbiologically evaluable population was a subset of the clinically evaluable population that had also microbiologically documented infections.

### Definitions of infections

Infections were defined according to the definitions used by the individual RCTs. Definitions of infections did not differ substantially between the RCTs included in the meta-analysis.

cIAIs were defined as intra-abdominal infections extending beyond site of origin, causing peritonitis or abscess formation, and thus requiring surgical intervention. APIs were defined clinically by the presence of fever (>38°C) or a white blood cell count >10 500/μl plus pelvic or abdominal or uterine pain or cramping or tenderness, or an imaging study suggesting pelvic abscess or infection; and a compatible prior gynecological history. cSSSIs were defined as skin and skin-structure infections with sufficient severity and signs of systemic illness, which is deeper, more indolent, and more severe than routine soft-tissue infections, such as perineal cellulitis or abscesses, extensive cellulitis, posttraumatic or postsurgical skin or soft-tissue infection, and lower-extremity infections in patients with diabetes mellitus.

### Analyzed outcomes

The primary efficacy outcome of this meta-analysis was clinical treatment success(defined as complete resolution or substantial improvement of symptoms and signs of cIAIs, cSSSIs, and APIs, or no further antimicrobial therapy and surgical intervention for infection was necessary) assessed at the test-of-cure (TOC) visit employed in each individual study. The secondary efficacy outcome was microbiological treatment success (defined as the eradication of baseline pathogens, or as presumed eradication based on the clinical outcomes when post-treatment cultures were not performed) and mortality. The analysis of efficacy outcomes were based on MITT and clinically evaluable populations in each included RCT.

The primary safety outcome of the meta-analysis was clinical and laboratory drug related adverse events (defined as any adverse events which deemed to be drug related, and observed during the treatment and the post-treatment period) in each included RCT.

### Data analysis and statistical methods

Statistical analysis were done with Review Manager version 5.0.17 (Cochrane Collaboration, Oxford, UK). We assessed heterogeneity of trial results by calculating a chi-square test of heterogeneity and the I^2 ^measure of inconsistency. The publication bias was assessed by examining the funnel plot. We used a fixed-effect model (FEM) by using the Mantel-Haenszel method for pooling odds ratios (ORs) and 95% confidence intervals (CIs) for all primary and secondary outcomes (including MITT, clinically evaluable, and microbiologically evaluable populations) throughout the meta-analysis unless statistically significant heterogeneity was found (p < 0.10 or I^2 ^> 50%), in which case we chose a random-effects model (REM) and used the DerSimonian and Laird method. Heterogeneity was investigated through subgroup analysis as defined above.

## Results

### Study selection process

**Figure 1:**
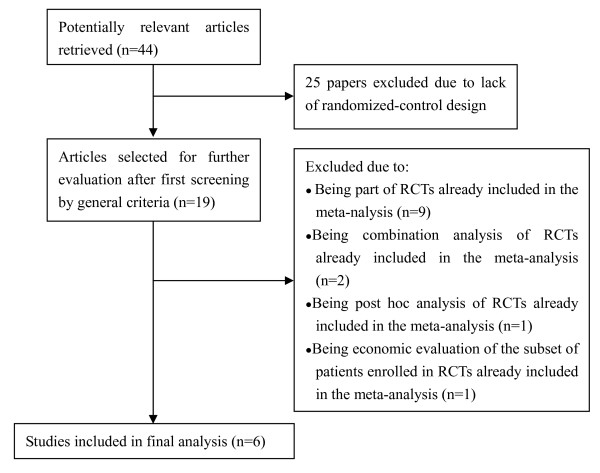
**Flow diagram of the randomized controlled trials (RCTs) reviewed**.

The flow diagram (Figure 1) showed the detailed screening and selection process for the trials included in this meta-analysis. The search was performed in PubMed, the Cochrane Central Register of Controlled Trials and Embase. We obtained 19 full papers from 44 studies for detail evaluation. Finally, we identified 6 RCTs, which fulfilled the criteria for inclusion in the meta-analysis.

### Study characteristics

The main characteristics of the 6 included RCTs (type of study design, characteristics of the included population, antimicrobial agents and doses used, concomitant antibacterial agents, number of enrolled patients and ITT patients, Jadad score) presents in Table 1. All of the included RCTs were performed exclusively in adult patients (three RCTs involved patients with cIAIs, 2 RCTs involved patients with cSSSIs and 1 RCTs involved exclusively adult female patients with APIs). High Jadad score (four RCTs had a score of 5, one had 4 and one had 3) indicated high quality of the RCTs included in the meta-analysis. We examined the funnel plot (SE of log OR plotted against ORs) to estimate publication bias, showing a symmetric inverse funnel distribution.

**Table 1:** Main characteristics of the trials included in the meta-analysis

Study	Type of study	Included population	Drug tested		concomitant antibacterial agents	Enrolled patients	Intention to treat	Jadad score
							
			Ertapenem	Piperacillin/tazobactm				
Dela Pena *et al.*	Multicentre open-label RCT	Hospitalized adults, ≥18 years-old, with cIAIs that extended beyond the wall of a hollow organ	Ertapenem 1 g once daily, i.v.(possible change to i.m. after 2 days of therapy)	Piperacillin/tazobactm 3.375 g i.v. q6h or 4.5 g i.v. q8h	Vancomycin or teicoplanin, for resistant Gram-positive pathogens	399	180vs190	3
Namias *et al.*	Multicentre double-blind RCT	Hospitalized adults,18-90 years-old, with presumptive or confirmed cIAIs	Ertapenem 1 g once daily, i.v., followed by a placebo every 6 h for three additional doses daily	Piperacillin/tazobactm 3.375 g i.v. q6h	Vancomycin if MRSA or enterococci isolated	500	247vs247	5
Roy *et al.*	Multicentre double-blind RCT	Females ≥16 years-old, With APIs, required ≥3 days of parenteral antimicrobial therapy	Ertapenem 1 g once daily, i.v., followed by a placebo every 6 h for three additional doses daily	Piperacillin/tazobactam 3.375 g i.v. q6h (adjusted in case of low creatinine clearance).	Vancomycin for resistant Gram-positive organisms, antifungals	450	216vs196	5
Solomkin *et al*.	Multicentre double-blind RCT	Hospitalized adults ≥18 years-old, with confirmed cIAIs	Ertapenem 1 g once daily, i.v., followed by a placebo every 6 h for three additional doses daily	Piperacillin/tazobactam 3.375 g i.v. q6h (adjusted for renal failure)	Vancomycin against enterococci or MRSA	633	323vs310	5
Graham *et al*.	Multicentre double-blind RCT	adults ≥18 years-old, with CSSSIs, required parenteral antimicrobial therapy	Ertapenem 1 g once daily, i.v., followed by a placebo every 6 h for three additional doses daily	Piperacillin/tazobactam 3.375 g i.v. q6h	Not permitted	540	274vs266	4
Lipsky *et al*.	Multicentre double-blind RCT	diabetes mellitus adult patients, with a foot infection that did not extend above the knee	Ertapenem 1 g once daily, i.v., followed by a placebo every 6 h for three additional doses daily	Piperacillin/tazobactam 3.375 g i.v. q6h	Vancomycin against enterococci or MRSA	639	295vs291	5

Regarding the characteristics of the population of the included RCTs involving patients with cIAIs, it was specifically reported that patients with severe infections (APACHE II score > 30[11,12,14]) were excluded. Most of the trials only included patients infected with pathogens susceptible to study antimicrobial treatments or with at least one pathogen being susceptible to study treatments in the case of polymicrobial infections, and excluded the patients with infections caused by pathogens known to be resistant to study treatments. In 5 of the overall 6 included RCTs, other than vancomycin or teicoplanin to treat infections caused by resistant gram-positive pathogens, concomitant use of other antimicrobial agents was not allowed. In the other one RCT [15], any concomitant use of other antimicrobial agents was not permitted.

All the patients in the ertapenem treatment arms received 1 g of ertapenem intravenously once daily. Except partial patients in trial performed by Dela Peta *et al *[11] received 4.5 g of piperacillin/tazobactam every 8 hours, all the other patients in piperacillin/tazobactam groups included in our meta-analysis received 3.375 g every 6 hours. The duration of administration of study treatments varied between studies, but no differences were found between the compared arms, in each individual RCT. We analysis all of the outcomes assessed at TOC visit which varied between the RCTs, with a minimum value of 10 days, to a maximum value of 4 weeks, after the completion of study treatments.

### Clinical treatment success and mortality

**Figure 2:**
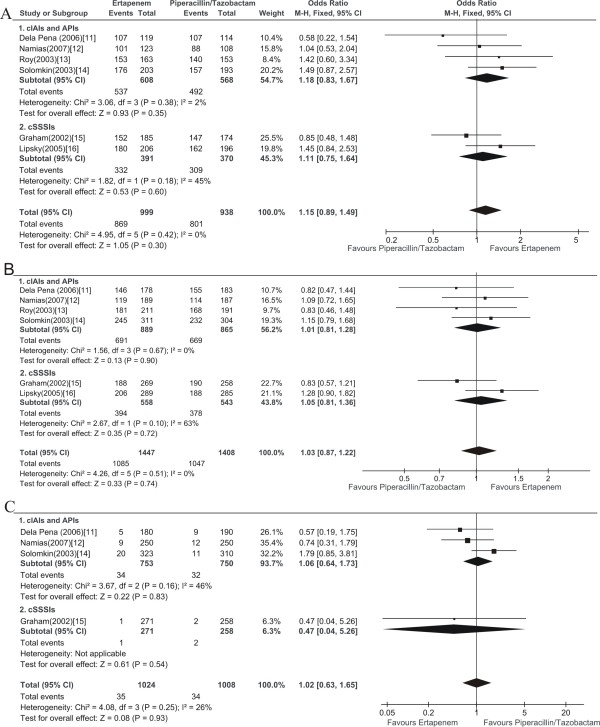
**Meta-analysis of clinical treatment success and mortality comparing ertapenem with piperacillin/tazobactam for complicated infections treatment at test-of-cure visit**. A, clinical treatment success analysis based on clinically evaluable population; B, clinical treatment success analysis based on modified intention to treat population; C, mortality analysis. Vertical line indicates no difference between ertapenem and piperacillin/tazobactam. The size of each square denotes the proportion of information given by each trial.

All of the 6 relevant RCTs provided the primary outcome (clinical treatment success) of the patients with complicated infections. The total clinical treatment success of ertapenem group was numerically higher than piperacillin/tazobactam group in clinically evaluable population at TOC visit, while no significant difference was found (1937 patients, FEM, OR: 1.15, 95% CI: 0.89-1.49, Figure 2A). The similar results were confirmed in the conservative MITT analysis in which patients with inadequate information or indeterminate outcomes were considered to be cases of treat failure (2855 patients, FEM, OR: 1.03, 95% CI: 0.87-1.22, Figure 2B).

Regarding the cIAIs and APIs or cSSSIs subgroup, there was also no significant difference in treatment success between the clinically evaluable patients treated with ertapenem, and those treated with piperacillin/tazobactam at TOC visit (cIAIs and APIs subgroup, 4 RCTs, 1176 patients, FEM, OR: 1.18, 95% CI: 0.83-1.67; cSSSIs subgroup, 2 RCTs, 761 patients, FEM, OR: 1.11, 95% CI: 0.75-1.64, Figure 2A). The conservative MITT analysis confirmed the above results (cIAIs and APIs subgroup, 4 RCTs, 1754 patients, FEM, OR: 1.01, 95% CI: 0.81-1.28; cSSSIs subgroup, 2 RCTs, 1101 patients, FEM, OR: 1.05, 95% CI: 0.81-1.36, Figure 2B).

Four of the 6 relevant RCTs provided mortality outcomes of the included patients. There was also no significant difference in mortality between the patients treated with ertapenem, and those treated with piperacillin/tazobactam at TOC visit (2032 patients, FEM, OR: 1.02, 95% CI: 0.63-1.65, Figure 2C).

The sensitivity analysis limited to double-blind RCTs, obtained similar finding regarding clinical treatment success in clinically evaluable population(5 RCTs, 1704 patients, FEM, OR: 1.21, 95% CI: 0.93-1.59) and MITT population (5 RCTs, 2494 patients, FEM, OR: 1.05, 95% CI: 0.88-1.26), and regarding mortality outcome (3 RCTs, 1662 patients, FEM, OR: 1.18, 95% CI: 0.68-2.03).

### Microbiological treatment success

**Figure 3:**
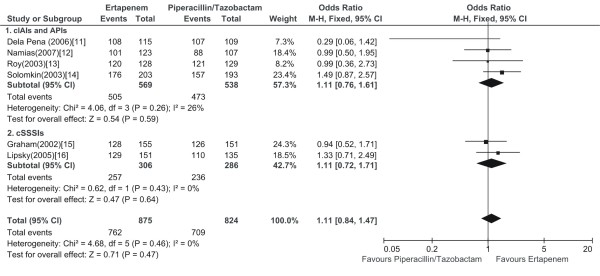
**Meta-analysis of microbiological treatment success comparing ertapenem with piperacillin/tazobactam for complicated infections treatment at test-of-cure visit**. The analysis based on microbiologically evaluable population. Vertical line indicates no difference between ertapenem and piperacillin/tazobactam. The size of each square denotes the proportion of information given by each trial.

All the 6 relevant RCTs provided the microbiological treatment success. The total microbiological treatment success of ertapenem group was numerically higher than piperacillin/tazobactam group in microbiologically evaluable population at TOC visit, while there was no significant difference (1699 patients, FEM, OR: 1.11, 95% CI: 0.84-1.47, Figure 3).

In the subgroup analysis including with cIAIs and APIs, no difference was found regarding microbiological treatment success between ertapenem and piperacillin/tazobactam treatment arm (4 RCTs, 1107 patients, FEM, OR: 1.11, 95% CI: 0.76-1.61, Figure 3). In the additional subgroup analysis including patients with cSSSIs, there was also no difference between compared treatment arms (2 RCTs, 592 patients, FEM, OR: 1.11, 95% CI: 0.72-1.71, Figure 3).

In the sensitivity analysis limited to double-blind RCTs, we got unchanged findings with the overall analysis regarding microbiological treatment success (5 RCTs, 1475 patients, FEM, OR: 1.17, 95% CI: 0.88-1.57).

### Drug related adverse events

Four of the 6 relevant RCTs provided the drug related clinical adverse events and 5 provided laboratory adverse events. The total drug related adverse events of ertapenem groups were slightly lower than piperacillin/tazobactam groups in safety evaluable population.

**Figure 4:**
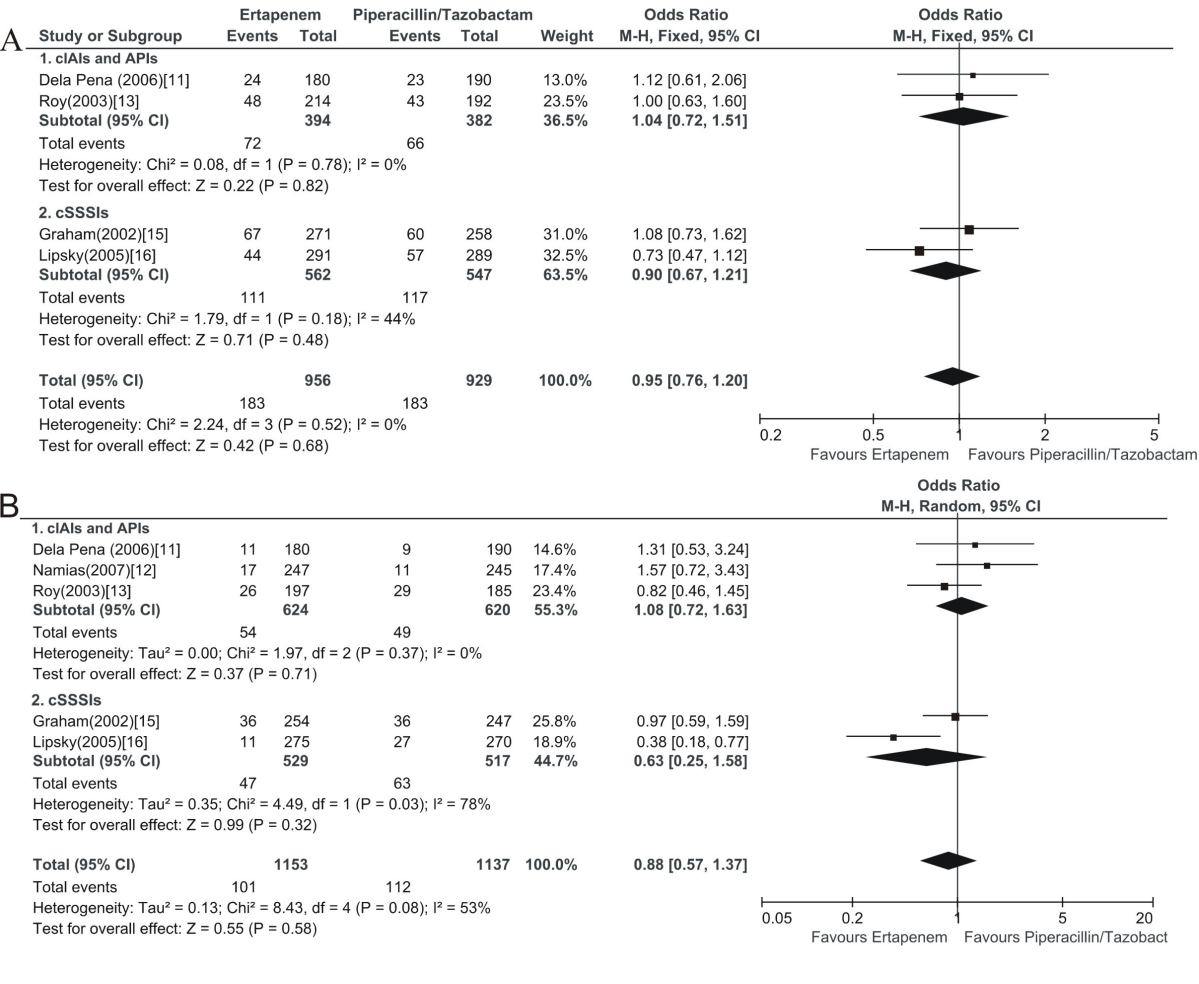
**Meta-analysis of drug related adverse events comparing ertapenem with piperacillin/tazobactam for complicated infections treatment during the treatment and the post-treatment period**. The analysis based on safety evaluable population. A, analysis of clinical drug related adverse events; B, analysis of laboratory drug related adverse events; Vertical line indicates no difference between ertapenem and piperacillin/tazobactam. The size of each square denotes the proportion of information given by each trial.

The most common drug related clinical adverse events in each treatment group (ertapenem vs. piperacillin/tazobactam recipients) were gastrointestinal, most commonly diarrhea, vomiting and nausea. These were generally of mild to moderate severity. No difference was found regarding drug related clinical adverse events between ertapenem and piperacillin/tazobactam treatment arms (1885 patients, FEM, OR: 0.95, 95% CI: 0.76-1.20, Figure 4A).

The most common drug related laboratory adverse events were increased liver transaminases (alanine aminotransferase and aspartate aminotransferase), increased serum alkaline phosphatase, and thrombocytosis. These elevations were generally mild to moderate and were transient, where follow-up information was available in the included RCTs. No other specific drug related adverse events occurred in >3% of patients in either treatment group. The analysis regarding drug related laboratory adverse events got similar results with the drug related clinical adverse events (2290 patients, REM, OR: 0.88, 95% CI: 0.57-1.37, Figure 4B).

The sensitivity analysis limited to double-blind RCTs, obtained similar findings with the overall analysis regarding either clinical adverse events (3 RCTs, 1515 patients, FEM, OR: 0.93, 95% CI: 0.72-1.19), or laboratory adverse events (4 RCTs, 1920 patients, REM, OR: 0.83, 95% CI: 0.50-1.37).

## Discussion

This systematic review with a meta-analysis compared the efficacy and safety of ertapenem with piperacillin/tazobactam in patients with complicated infections including cIAIs, APIs and cSSSIs. The main result of this meta-analysis with regard to the primary efficacy outcome (clinical treatment success) suggested that no difference existed between the treatment arms (Figure 2). The secondary outcome analysis produced finding consistent with the primary outcome (Figure 2, 3). In the ertapenem treatment arms, the drug related clinical adverse events mainly were gastrointestinal and the drug related laboratory adverse events consisted primarily of elevations in liver enzymes (transaminases and alkaline phosphatase) and thrombocytosis. The safety analysis regarding to the drug related clinical and laboratory adverse events both proved no difference between the compared treatment arms (Figure 4). Likewise, post hoc analysis of trials comparing ertapenem treatment with piperacillin/tazobactam, in patients with cIAIs, APIs and cSSSIs, also have not revealed any significant differences, regarding the efficacy and safety outcomes examined [20].

An earlier meta-analysis performed by Falagas *et al *[21]had provided evidence that ertapenem was comparable to other recommended antimicrobial regimens (piperacillin/tazobactam, ceftriaxone plus metronidazole and ticarcillin/clavulanic acid) for the treatment of cIAIs. The focus of our meta-analysis was to compare the efficacy and safety of ertapenem with piperacillin/tazobactam, a well-established therapeutic agent, in patients with complicated infections. APIs, cSSSIs and cIAIs are most often polymicrobial infections. They have similarities both in aetiological organisms involving a mixture of gram-positive, gram-negative anerobic and anaerobic organisms such as staphylococcus, streptococci, enterococci, Enterobacteriaceae, anaerobic coccobacilli, anaerobic bacilli, and in empiric antimicrobial treatment that requires parenteral coverage of a broad spectrum of potential pathogens including an extended-spectrum cephalosporin plus an agent active against anaerobes including an extended-spectrum cephalosporin plus an agent active against anaerobes, such as metronidazole or clindamycin, or a β-lactam/β-lactamase inhibitor combination. Piperacillin/tazobactam, a β-lactam/β-lactamase inhibitor combination agent, has been well studied and is approved in the treatment of complicated infection including cIAIs, APIs, and cSSSIs [22]. So in the present meta-analysis, we included RCTs performed on APIs and cSSSIs, in addition to those performed on cIAIs.

It should be pointed out that ertapenem, although highly active *in vitro *against methicillin-susceptible staphylococci and most clinically important Enterobacteriaceae, streptococci and anaerobes which are the most common causal pathogens in cIAIs, APIs and cSSSIs, is not against non-fermentative gram-negative bacilli, such as *Pseudomonas aeruginosa*, which are important nosocomial pathogens, particularly in intensive care units, or enterococci, which in the setting of a polymicrobial infection are of questionable pathogenicity[23,24]. Notably, in our meta-analysis ertapenem had a comparable efficacy (Figure 2, 3) to piperacillin/tazobactam, an agent possessing antienterococcal and good antipseudomonal activity. Clinical and microbiologic success rates for patients infected with *Pseudomonas aeruginosa *or enterococci were generally similar in each relevant RCT, and the high favorable response rates implied routine enterococcal or pseudomonal coverage was not essential, which was consistent with previous study [25-27] and the current recommendations from the Surgical Infection Society [28] and the Infectious Disease Society of America [29]. However, this finding should be interpreted with caution, given that it is based on a rather small total number of patients in our meta-analysis.

Although the present evidence suggests ertapenem had similar efficacy and safety profile with piperacillin/tazobactam in the treatment of complicated infections such as cIAIs, APIs and cSSSIs, several unique characteristics make it as an alternative to piperacillin/tazobactam. Firstly, ertapenem may mitigate the emergence of resistant strains. Previous studies indicated that there were fewer instances of emergence of resistant Enterobacteriaceae and extended-spectrum β-lactamase producing Enterobacteriaceae in the colonizing bowel flora of ertapenem-treated patients with cIAIs than receiving piperacillin/tazobactam or ceftriaxone plus metronidazole, and there were no differences between groups in the prevalence of imipenem-resistant *Pseudomonas *in the colonizing bowel flora after completion of therapy [30,31]. This may be attributed to the limited development of microbial resistance to carbapenems and the targeted spectrum of ertapenem [32]. Secondly, once-daily dosing of ertapenem may offer benefits compared with agents that require multiple dosing or combination therapy, including patient convenience and comfort, and a lower risk of medication errors [32]. Thirdly, compared with piperacillin/tazobactam given four times daily i.v., ertapenem given once daily i.v. was associated with lower drug and supply costs and less time and labor devoted to the preparation and administration of i.v. therapy[33].

In contrast to the earlier meta-analysis [21], we examined 6 high quality RCTs (Four RCTs had a Jadad score of 5, one had 4 and one had 3), focused on direct comparison of treatments with ertapenem and piperacillin/tazobactam as monotherapy, precluding the interference of other drugs, and included RCTs performed on APIs and cSSSIs in addition to cIAIs. The efficacy and safety outcome of our meta-analysis were defined similarly in the individual trial. Furthermore, the same administration route and doses of ertapenem (1 g once daily, i.v.) was used to compare with recommended dose of piperacillin/tazobactam in patients of the included RCTs. So the similar treatment schedule and evaluation criteria of the included trials provided greater statistical confidence for our meta-analysis.

The meta-analysis is not without limitations. First, the clinical treatment success analysis based on the data defined as cure or improvement of signs and symptoms may not be as accurate as complete cure. Four of the 6 overall included studies provided mixed data regarding the outcome of clinical treatment success. This may hinder a meaningful separate meta-analysis of complete cure. Second, in 3 RCTs performed on cIAIs, patients with severe infections (APACHE II score > 30 or of a life-threatening degree) were excluded, so the finding of this meta-analysis should be interpreted with caution on severe infections. Third, most included RCTs excluded patients with infections caused by pathogens known to be resistant to study treatment, which may interfere with the finding. Finally, all of the 6 included trials, including the one nonblinded study, were supported by the branding pharmaceutical company of ertapenem, a factor that might generate bias in the assessment of outcomes. Nevertheless, the sensitivity analysis limited to double-blind RCTs was performed in our meta-analysis, resulting in findings consistent with those of the primary analysis.

## Conclusion

Despite the limitations, the efficacy of ertapenem 1 g once daily was statistically equivalent to recommended dose of piperacillin/tazobactam, and was generally well tolerated. The present evidence suggests that the clinical use of ertapenem is associated with decreased emergence of antimicrobial resistance and lower costs. The once-daily dosing of ertapenem may be a useful alternative for combination and/or multidosed antibiotic regimens for the empiric treatment. However, there is rather limited evidence for the efficacy of ertapenem compared to piperacillin/tazobactam, in patients with severe complicated infections caused by one or more pathogens.

## Abbreviations

cIAIs: complicated intra-abdominal infections; APIs: acute pelvic infections; cSSSIs: complicated skin and skin-structure infections; RCTs: randomized controlled trials; ITT: intention to treat; MITT: modified intention to treat; TOC: test-of-cure; FEM: fixed-effect model; REM: random-effects model; OR: odds ratios; CI: confidence intervals.

## Competing interests

The authors declare that they have no competing interests.

## Authors' contributions

MMA and ZZ conducted the literature search and data analysis. All authors wrote and reviewed the manuscript.

## Pre-publication history

The pre-publication history for this paper can be accessed here:

http://www.biomedcentral.com/1471-2334/9/193/prepub
